# Supplementary material for: Whole-genome DNA methylomes of tree shrew brains reveal conserved and divergent roles of DNA methylation on sex chromosome regulation
Source: BMC Biol. 2024 Nov 28;22:277. doi: 10.1186/s12915-024-02071-0 (PMC11603898; doi:10.1186/s12915-024-02071-0)
Supplement: Supplementary file 1 — Additional File 1: Table S1. Overview of samples from 3 male and 3 female tree shrews and the processing of whole-genome bisulfite sequencing (WGBS) data. Fig. S1. Overview of DNA methylation data. Fig. S2. DNA methylation patterns near gene region. Fig. S3. Relationships between DNA methylation and gene expression for promoters and gene bodies for the X chromosome and a representative autosome (Chromosome 8) in male and female tree shrews. Fig. S4. Sex-specific read-depth on the X chromosome. Fig. S5. Comparisons of promoter and gene body DNA methylation in females and males for chromosome 8. Fig. S6. Global patterns of female X hypomethylation in tree shrews. Fig. S7. Differentially methylated regions (DMRs) between female and male tree shrews in the prefrontal cortex. Fig. S8. The observed hypomethylation of female X chromosomes is not due to biases arising from differing read depths between female (XY) and male (XX) samples. Fig. S9. A comparison of CpG O/E ratios among humans, koalas, and tree shrews. Fig. S10. Overview of differentially expressed genes between the male and female tree shrews. Fig. S11. A conserved DNA methylation pattern near the Xist gene. Fig S12. The putative Sry gene region. Fig. S13. Information on CpG site coverage in our data and the reproduction of results using highly covered CpG sites. [file 12915_2024_2071_MOESM1_ESM.docx]

# Supplementary data

**Table S1.** Overview of samples from 3 male and 3 female tree shrews and the processing of whole-genome bisulfite sequencing (WGBS) data.

#
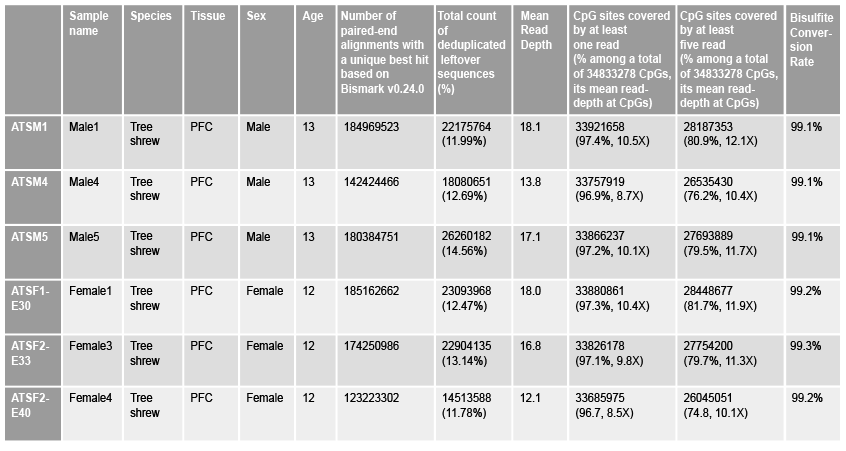


#
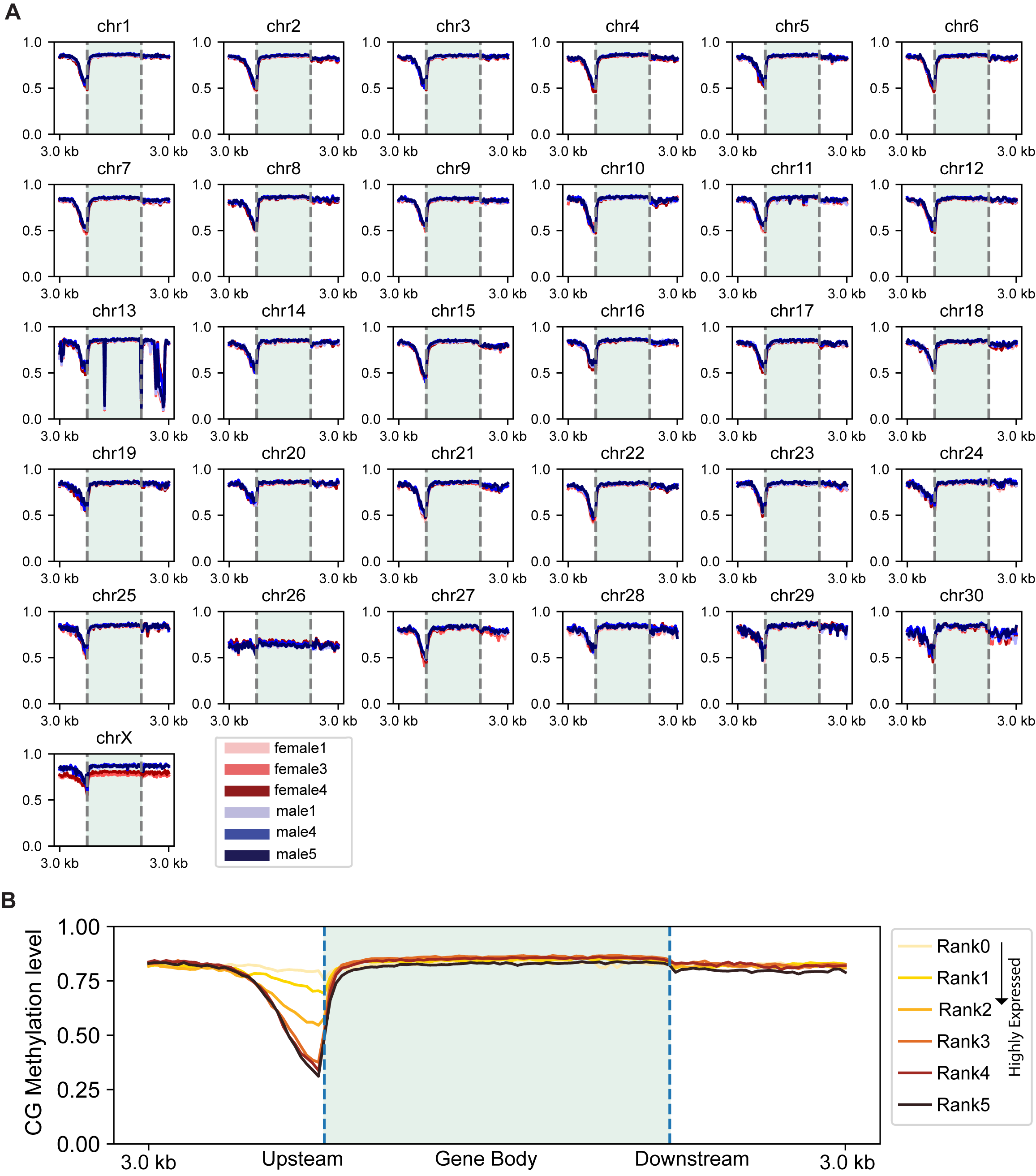


**Figure S1.** Overview of DNA methylation data. (A) Mean (Weighted) DNA methylation of protein-coding genes (22,741 genes) near gene body (highlighted in green), TSS, and gene end regions for chromosomes 1–30 and the X chromosome. The X-axis represents discrete bins ranging from 3kb upstream including promoters through the gene body region to 3kb downstream of genes while the Y-axis indicates the DNA methylation level. Notably, chromosomes 13 and 26 exhibit distinct patterns compared to the other chromosomes and are excluded from further analysis. Chromosome 26 displays a significant drop near the end of genes, while chromosome 13 consistently exhibits markedly lower methylation levels across all genomic contexts. (B) Mean DNA methylation near gene bodies in 5 groups of protein-coding genes with different averaged transcription levels. The rank0 group represents genes with no expression, and the rank5 group represents the genes with the highest expression level. A distinct negative correlation between methylation levels and gene expression levels was observed near the TSS region.


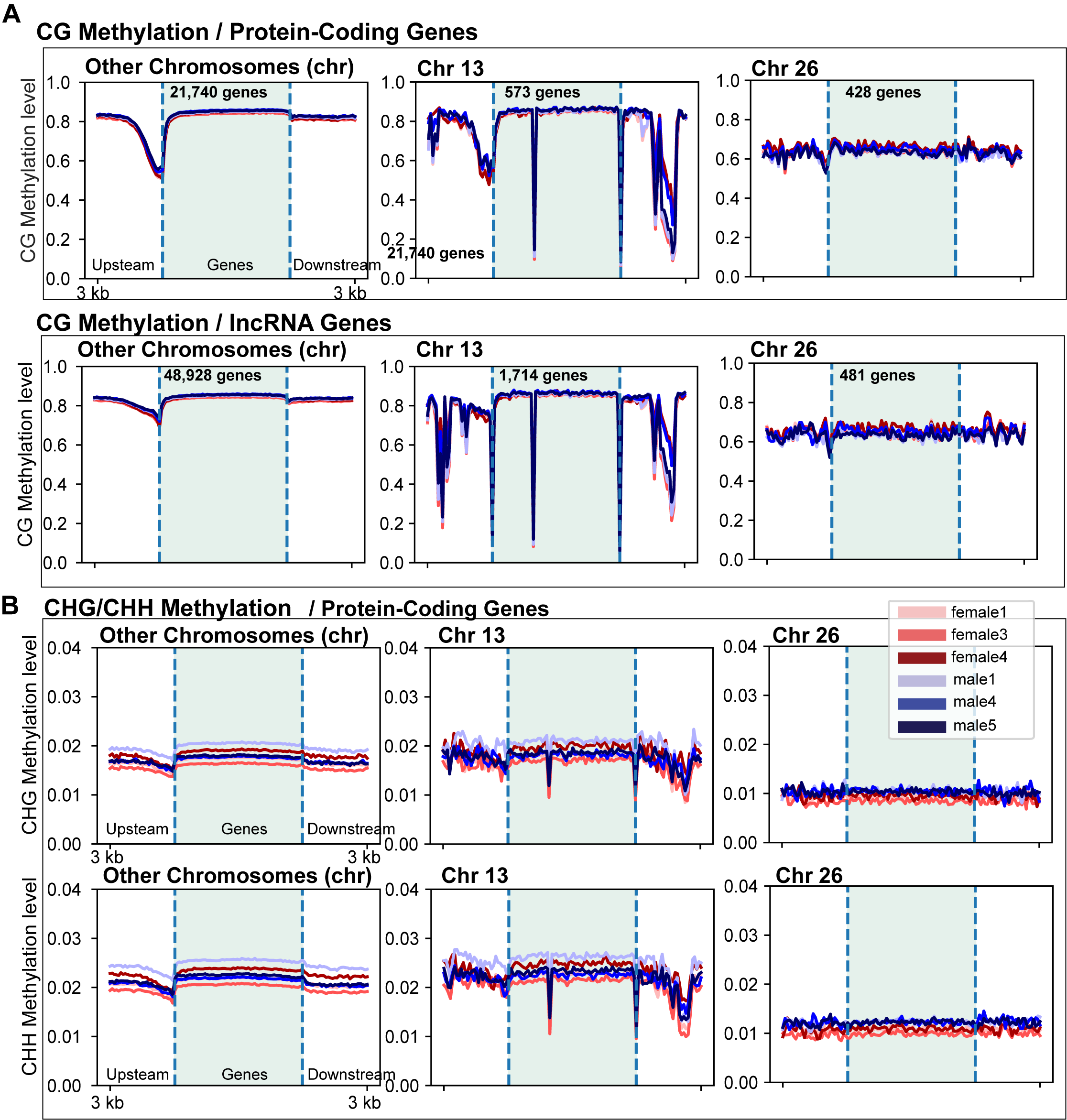


**Figure S2.** DNA methylation patterns near gene region. (A) Mean DNA methylation levels within gene bodies, 3kb upstream, and downstream regions for protein-coding genes and lncRNA genes on chromosome 13, chromosome 26, and other chromosomes. (B) Non-CG DNA methylation patterns near gene regions of chromosome 13 and chromosome 26, and other chromosomes. They show an irregular DNA methylation pattern in chromosome 13 and a comparatively lower DNA methylation level in chromosome 26. Furthermore, protein-coding genes exhibit a more pronounced methylation drop near the TSS compared to lncRNA genes.Top of Form Non-CG DNA methylation exhibits modest variations across diverse genomic contexts, displaying a subtle decrease near the Transcription Start Site (TSS).


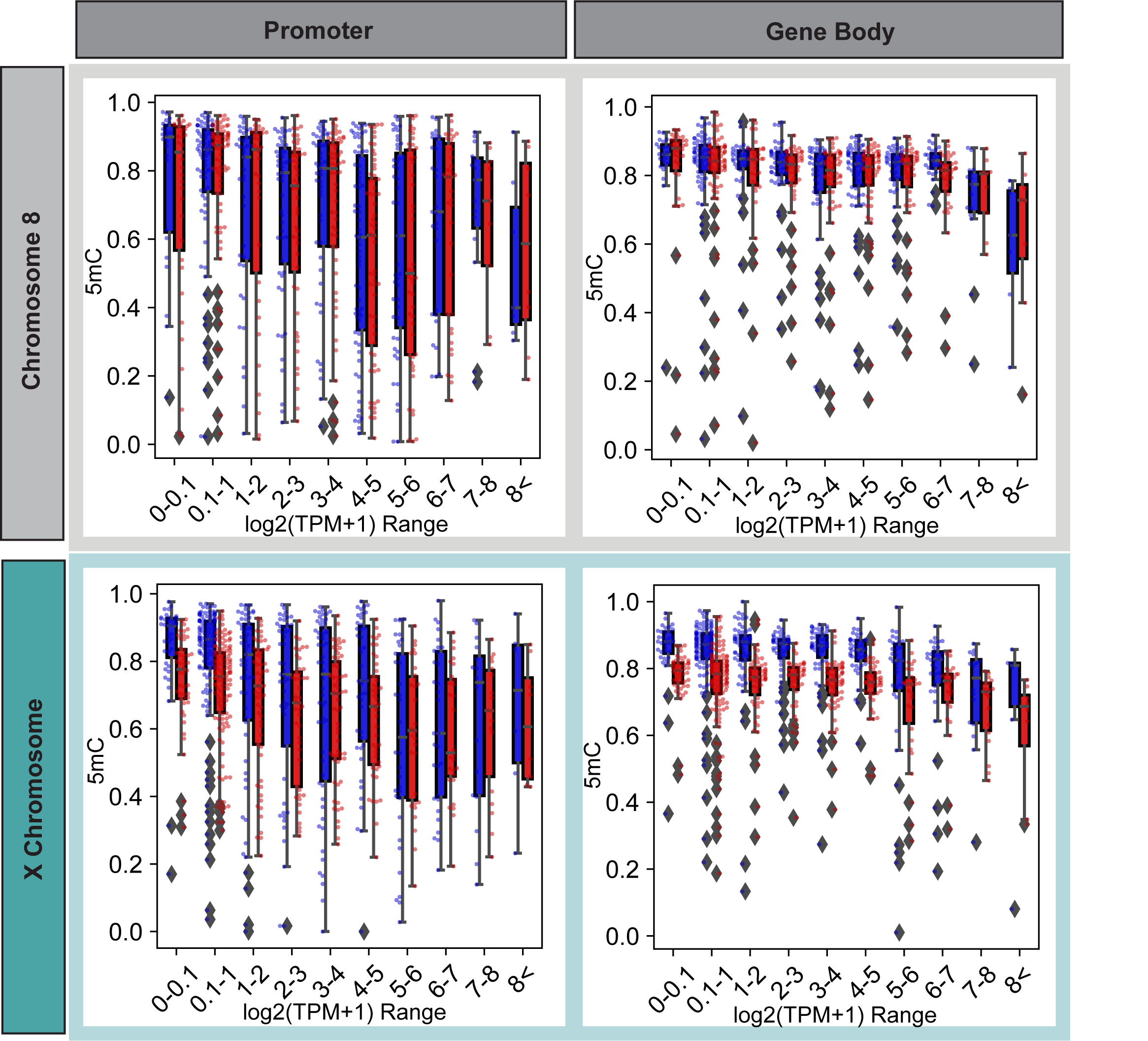


Figure S3. Relationships between DNA methylation and gene expression for promoters and gene bodies for the X chromosome and a representative autosome (Chromosome 8) in male and female tree shrews. Mean DNA methylation levels in these regions were averaged for females (in red) and males (in blue). The protein-coding genes are divided into 10 bins based on their expression values. Consistent negative correlations between promoter methylation levels and expression levels were observed across both chromosomes and in both females and males. A bell-shaped correlation was once again noted in the gene body regions. While data from chromosome 8 do not exhibit distinction between the females and males, the X chromosome clearly demonstrates female hypomethylation for both promoters and gene bodies.


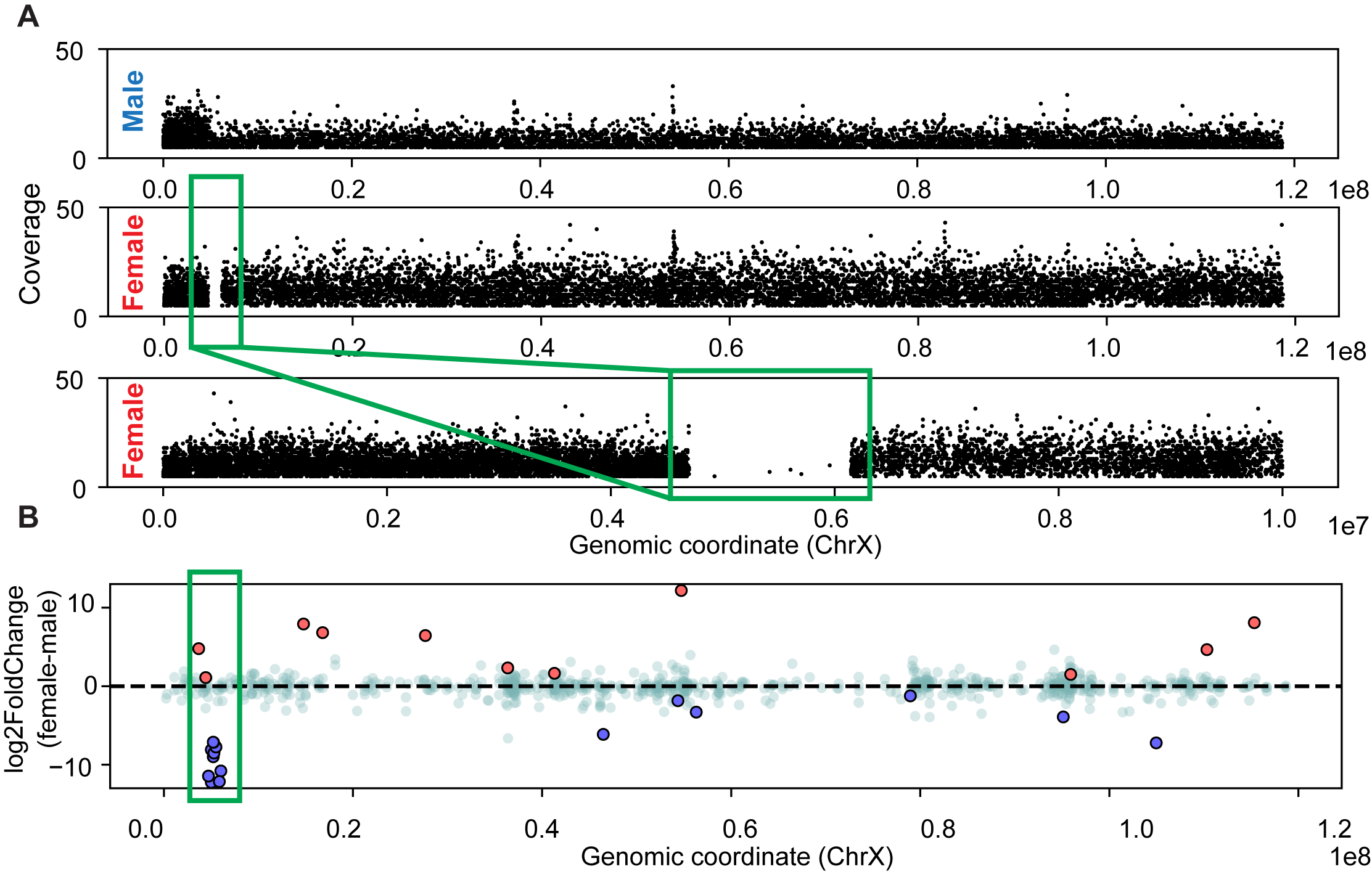


**Figure S4.** Sex-specific read-depth on the X chromosome. (A) A comparison of sequencing coverage between females and males at each CpG site across the X chromosome reveals a specific region (Genomic coordinates 4542400-6144400, highlighted in green) with a complete absence of read counts in the female sample. (B) Furthermore, the genes within this region exhibit male-specific expression patterns, leading us to define it as a segment of the Y chromosome mistakenly annotated within the X chromosome. Consequently, we have excluded both the CpG sites and 74 genes located within this region from our analysis of the X chromosome.


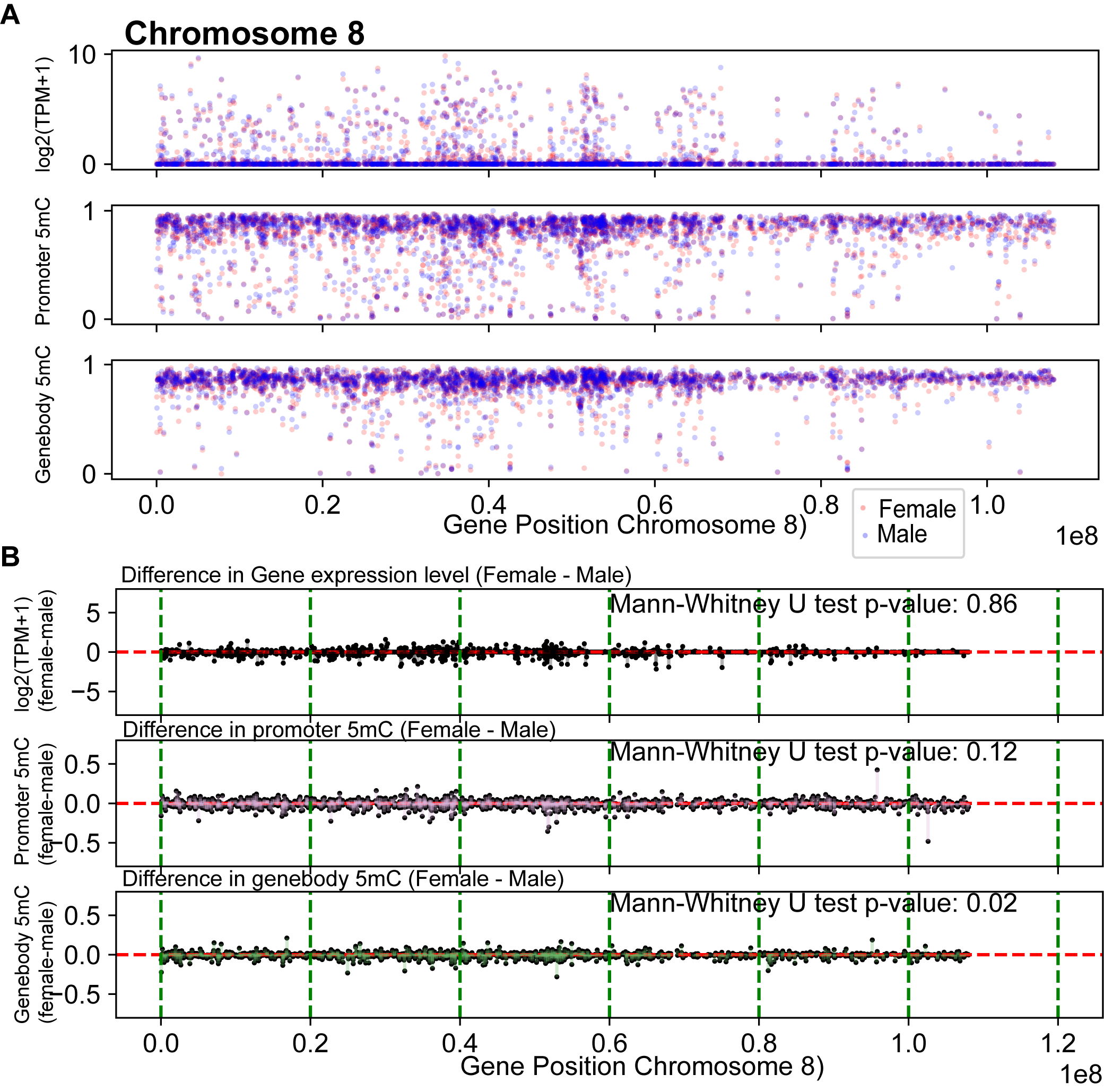


**Figure S5.** Comparisons of promoter and gene body DNA methylation in females and males for chromosome 8. The distribution of genes (2341 genes including both protein-coding genes and lncRNA genes) across chromosome 8, their expression levels, promoter DNA methylation levels, and gene body DNA methylation level of females (red) and males (blue) (A). Also, the differences between females and males (B). The levels were averaged in female and male samples. It exhibited no distinct differences of DNA methylation levels between females and males, compared to the chromosome X (Figure 2D).


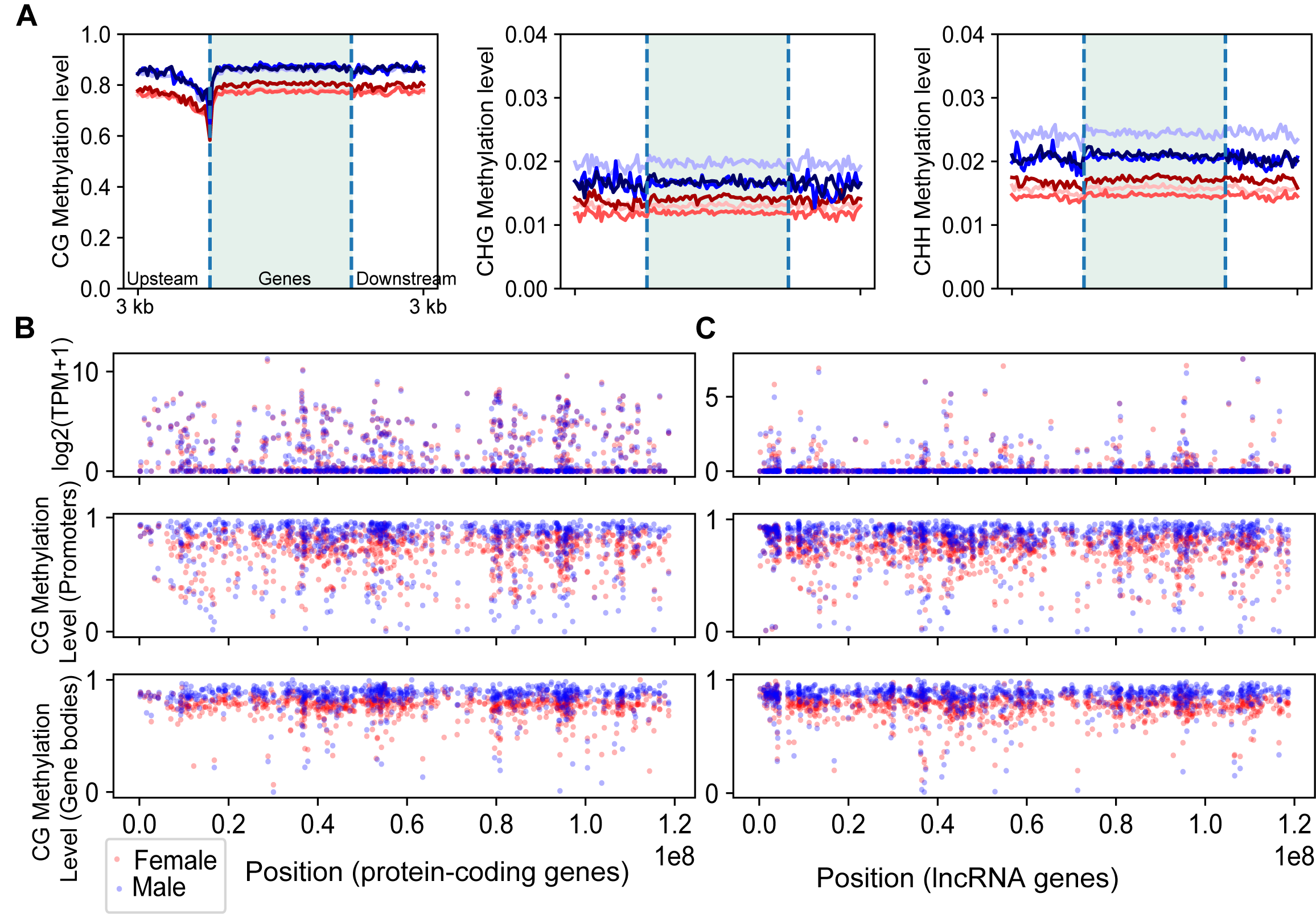


**Figure S6.** Global patterns of female X hypomethylation in tree shrews. (A) The weighted DNA methylation level near genes in the X chromosomes (1856 genes) of females (reds) and males (blues), showed female hypomethylation in CG context, CHH context, and CHG context. (B) The distribution of 823 protein-coding genes across the X chromosome, their expression levels, promoter DNA methylation levels, and gene body DNA methylation level of females (red) and males (blue) (C) Same analysis for 1058 lncRNA genes.


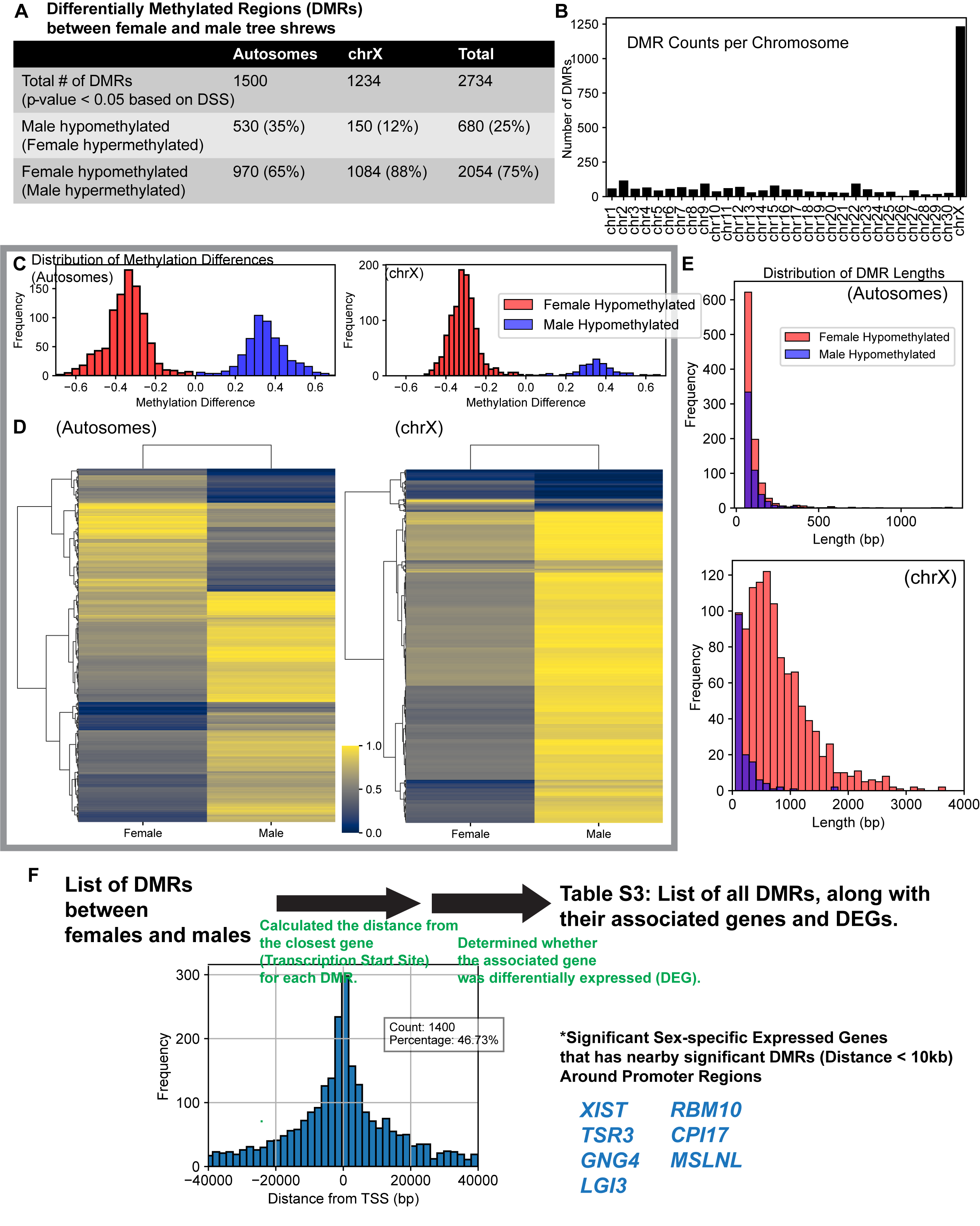


**Figure S7.** Differentially methylated regions (DMRs) between female and male tree shrews in the prefrontal cortex. (A) Counts of detected DMRs based on DSS with p-value < 0.05. (B) A high proportion of DMRs between females and males are located on the X chromosome (45%). (C) Distribution of DNA methylation differences between females and males (female-male) for DMRs, comparing autosomes and the X chromosome. In both autosomes and the X chromosome, there are more female hypomethylated DMRs than male hypomethylated DMRs. On the X chromosome, 88% of DMRs are female hypomethylated. (D) Heatmap showing DNA methylation levels in these DMRs, comparing females and males. (E) The length of DMRs detected on autosomes tends to be shorter than those on the X chromosome. These results are consistent with our finding that the female X chromosome is globally hypomethylated compared to the male X chromosome, with a total DMR length of 890,424 bp (85%) on the X chromosome and 155,177 bp (15%) on autosomes. (F) We calculated the distance between DMRs and the closest gene's transcription start site (TSS) (Table S3) to identify associated genes. Overall, DMRs tend to be positioned close to TSSs, with 46% within a 10,000 bp distance. Some DMRs were located near significantly sex-specific expressed genes. A list of sex-specific expressed genes (significant DEGs) that are potentially regulated by sex-specific DMRs near the TSS region is provided.


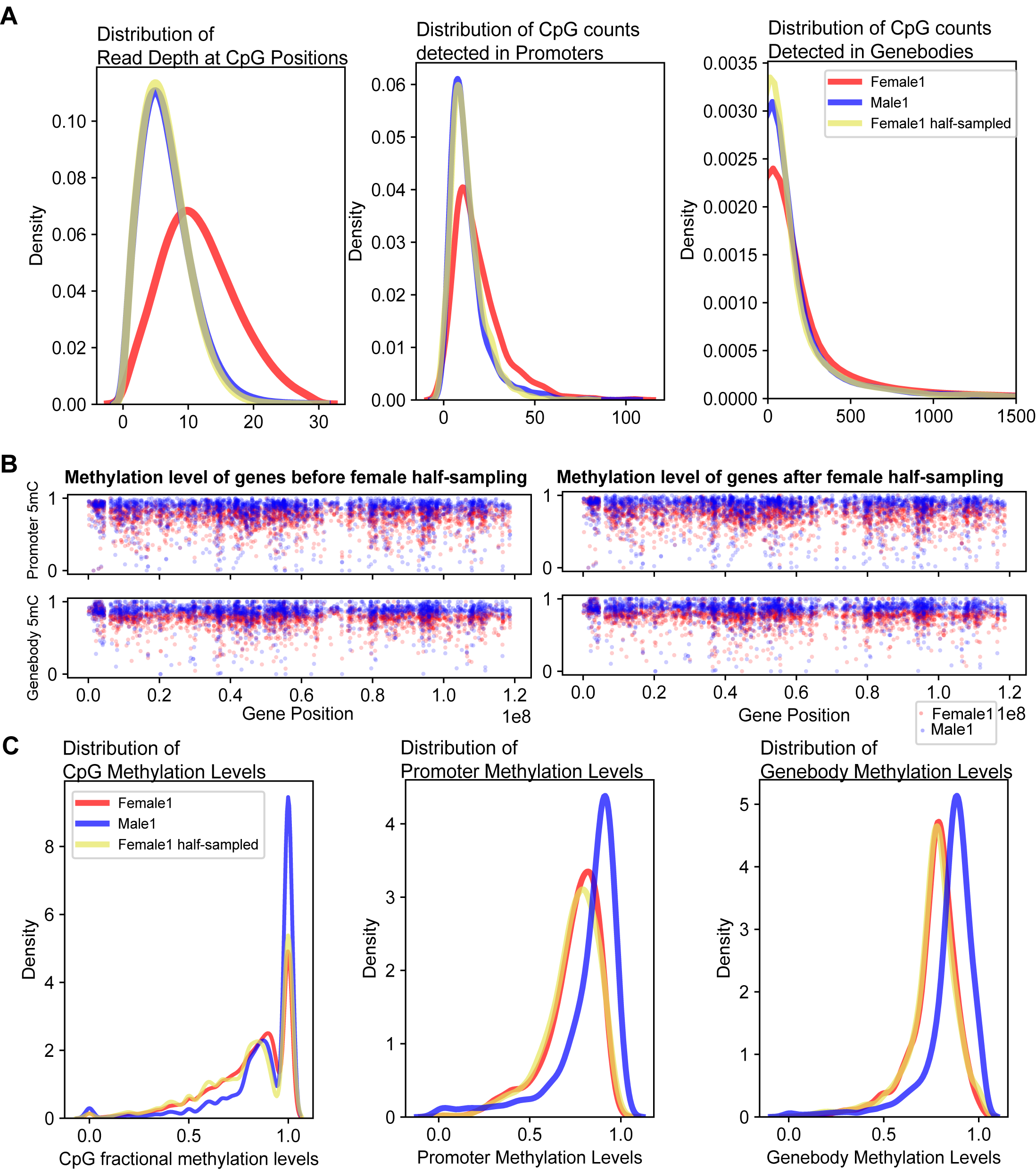


**Figure S8.** The observed hypomethylation of female X chromosomes is not due to biases arising from differing read depths between female (XY) and male (XX) samples. We equalized the sequence depth of X chromosomes by randomly sampling half of the reads from a female sample. (A) We verified this method by comparing the read depths at individual CpG sites and the number of CpG counts detected in promoter or gene body regions between a female sample and its half-sampled counterpart, in comparison to a male sample. (B) Hypomethylation of female X chromosomes is consistent across the entire X chromosome both before and after equalizing sequence depth. (C) Hypomethylation of female X chromosomes, in comparison to male X, is consistently observed irrespective of sequence depth for single CpGs and methylation levels in promoters and gene bodies.Top of Form

**
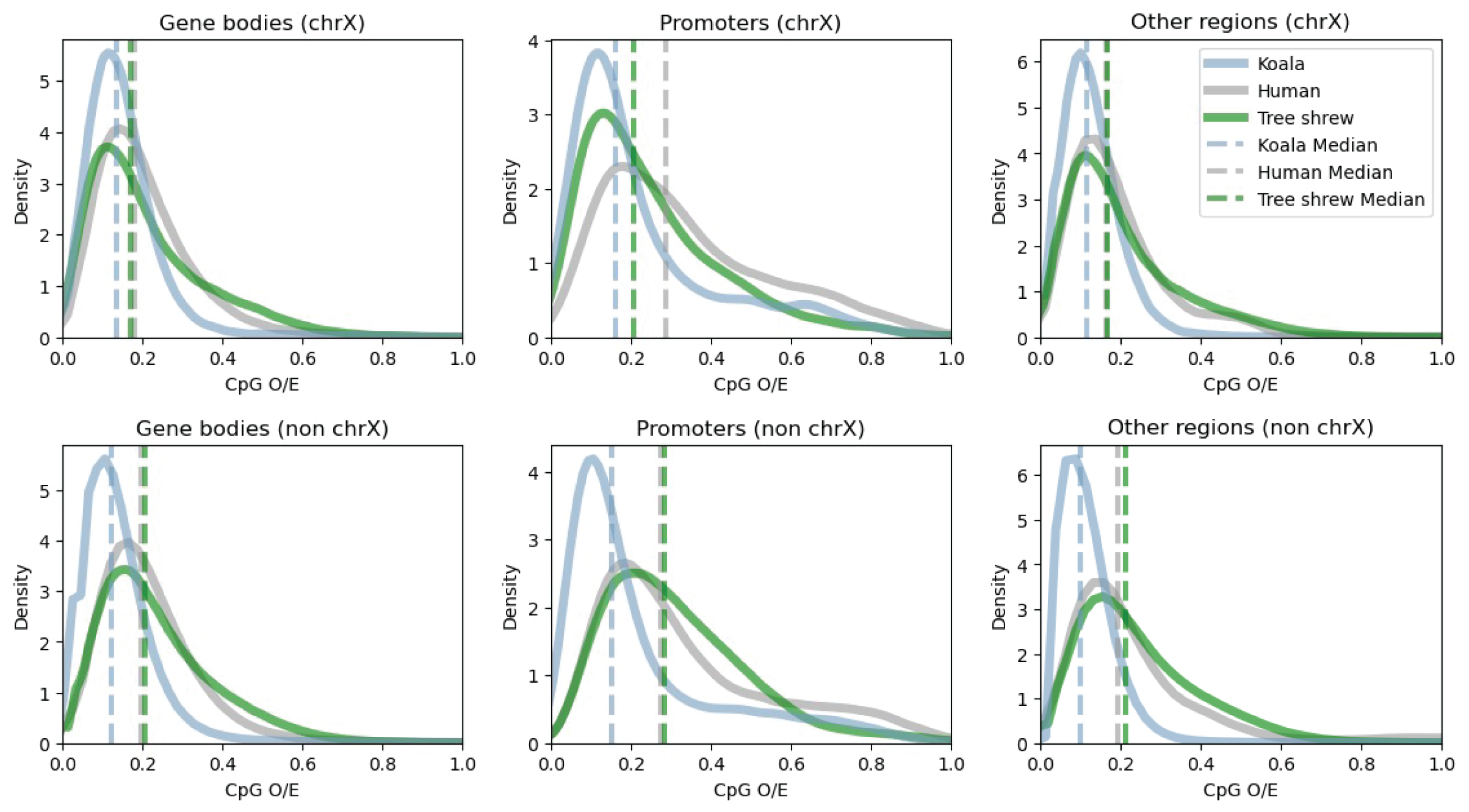
**

**Fig. S9.** A comparison of CpG O/E ratios among humans, koalas, and tree shrews. The CpG O/E ratio is calculated for 1000 bp-sized windows across the genome of each species, with each window annotated as a gene body, promoter, or other region. It is observed that tree shrews and humans have a closer CpG O/E ratio across the genome compared to that of koalas. However, a unique pattern is observed in the promoter regions of chromosome X, where similarity is noted between koalas and tree shrews compared to humans. This observation explains the similar female X hypomethylation pattern in tree shrews and humans, as the degree of female hypomethylation compared to males in promoters depended on the number of CpG sites (Fig. 3E, F)


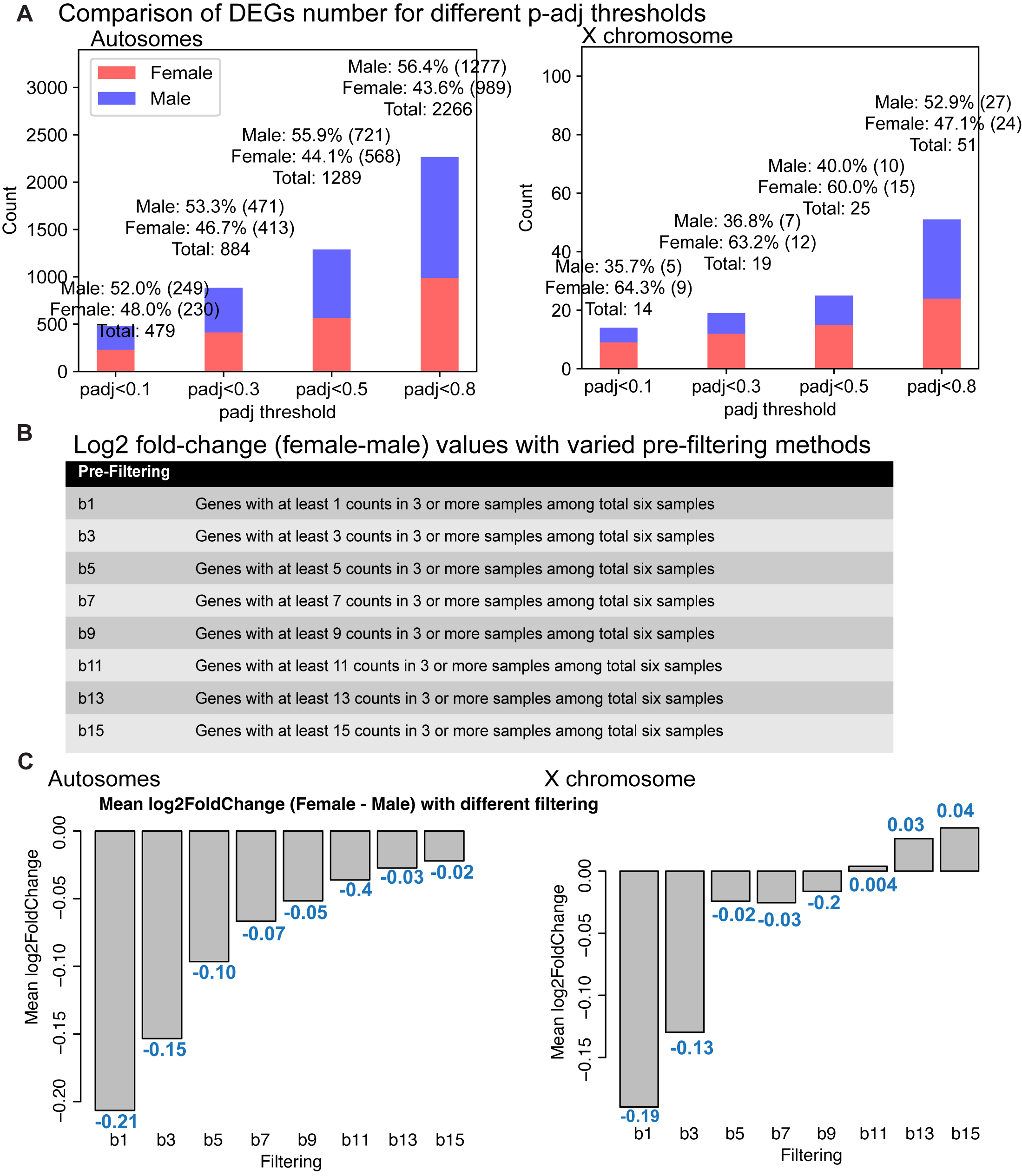


**Figure S10.** Overview of differentially expressed genes between the male and female tree shrews. (A) Number of sex-specifically expressed genes detected by DESeq2 across various adjusted p-value thresholds for autosomes and the X chromosome. A consistent pattern of more genes up-regulated in males compared to females is observed in autosomes.

(B-C) Average Log2 fold-change (female-male) values calculated from DESeq2 analysis with varied pre-filtering methods for autosomes (left) and the X chromosome (right). (B) Various pre-filtering methods employed in the analysis. (C) Plot illustrating the mean log2 fold change with different filtering stringency.


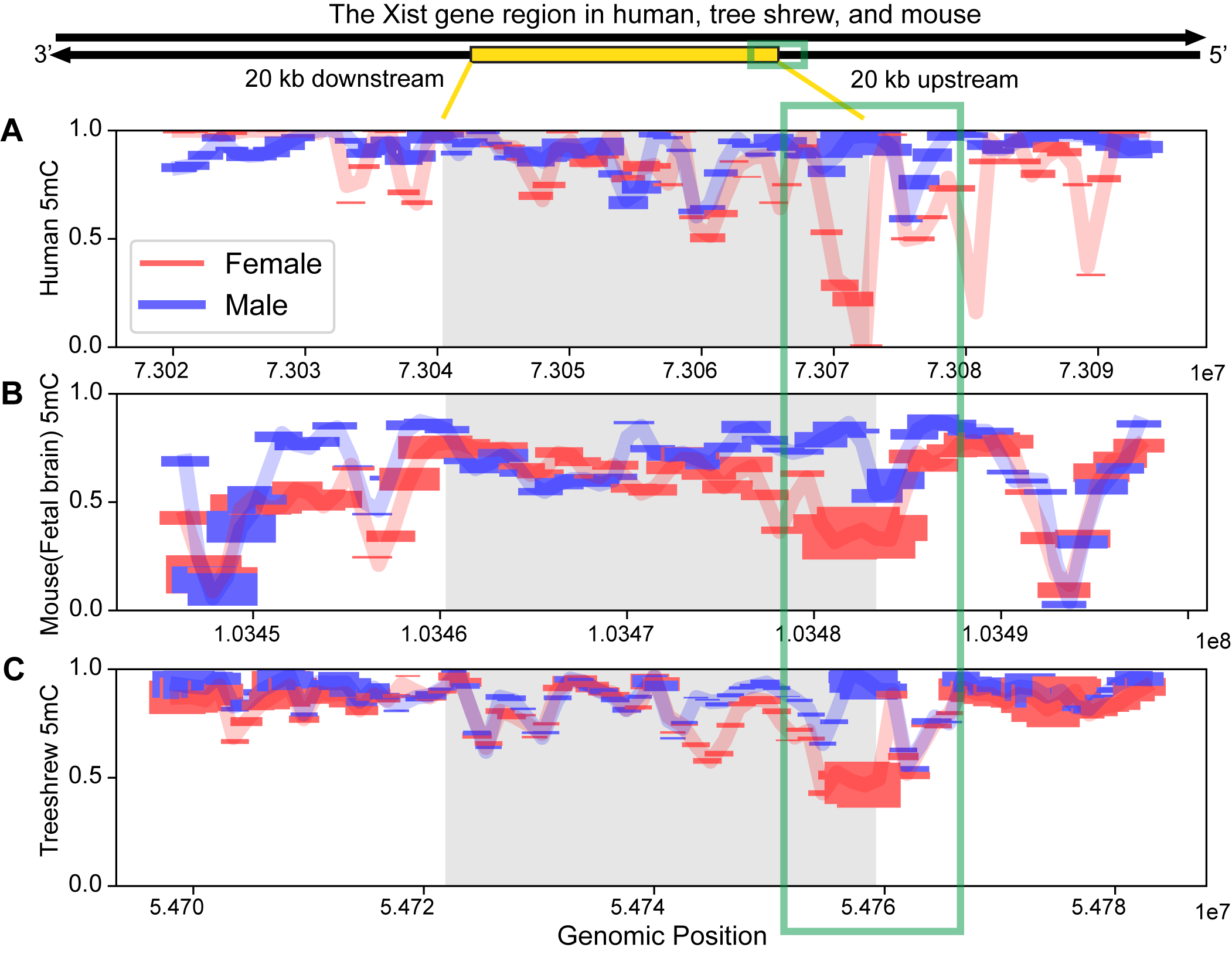


Figure S11. A conserved DNA methylation pattern near the *Xist* gene. WGBS data from two human brains, six tree shrew brains, and four mouse fetal brains were analyzed (See Method), with a focus on comparisons between females (red) and males (blue). The observed female hypomethylation near the 5’ end (green box) of the gene emerges as a conserved feature across these different species.

Bottom of Form


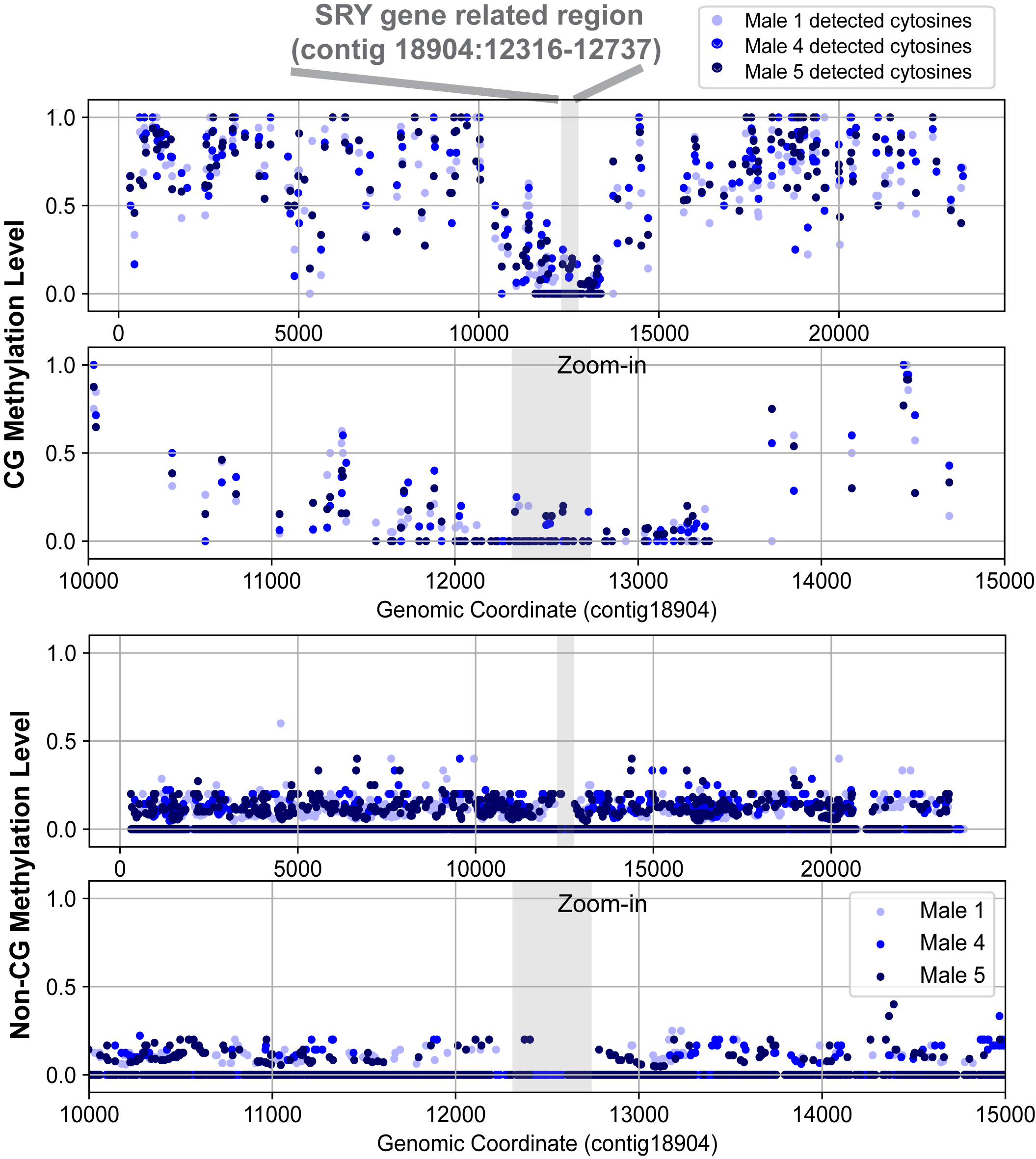


**Fig S12.** The putative *Sry* gene region. The target locus is highlighted in grey on contig18904, identified through BLASTN analysis of the human and rhesus monkey *Sry* against the tree shrew genome, along with the methylation levels of individual CpGs detected in CG and non-CG contexts in male samples. Extreme hypomethylation is evident in the *Sry* gene regions and its nearby regions (in both CG and non-CG contexts), and this pattern is consistently observed in all three male samples. This extreme hypomethylation is potentially related to the absence of expressed transcripts detected in the transcriptome data from male tree shrew prefrontal cortex samples.


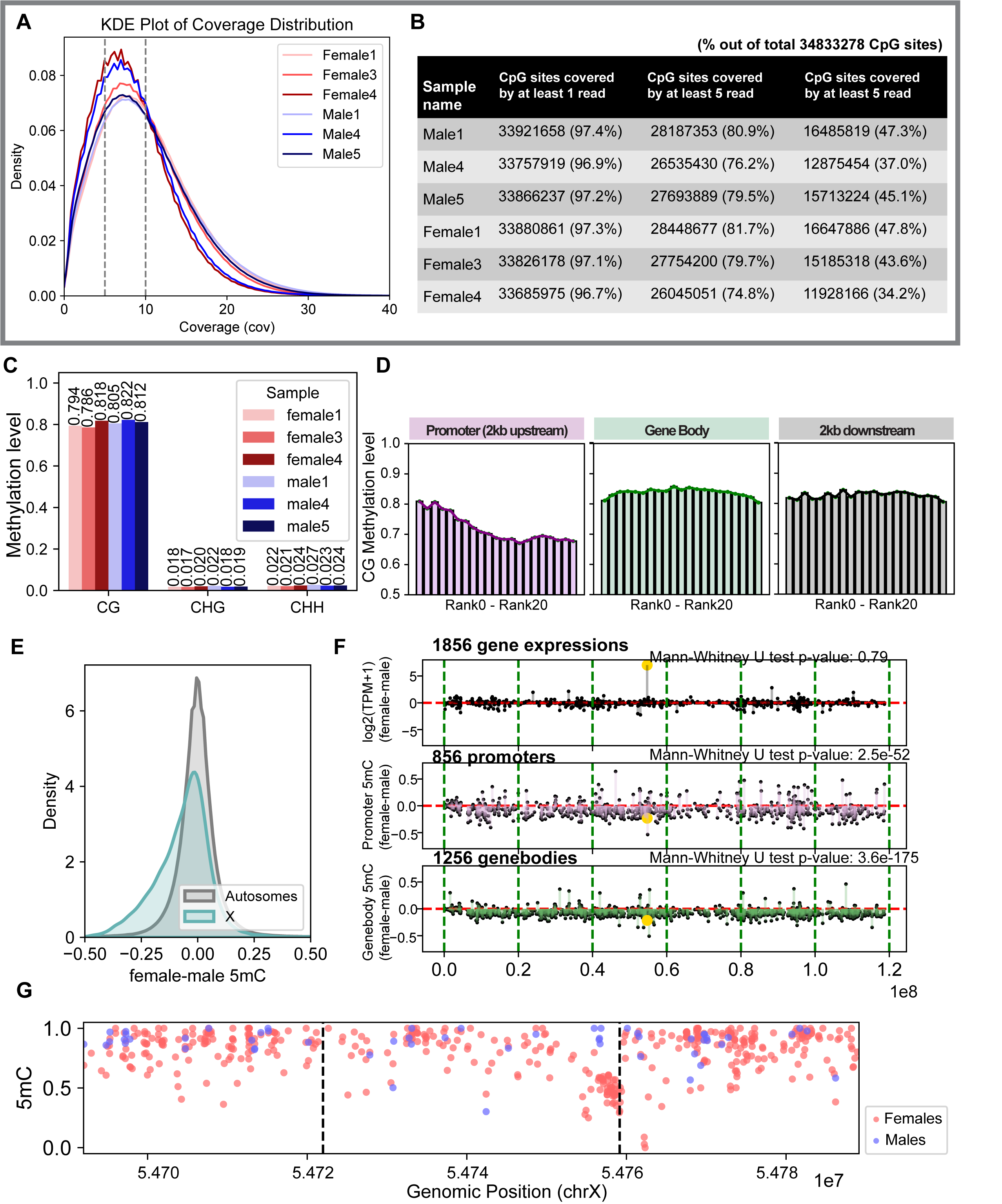


**Figure S13.** Information on CpG site coverage in our data and the reproduction of results using highly covered CpG sites. (A) The distribution of read coverage at CpG sites in each sample. (B) The number and proportion of CpG sites covered at different read coverage thresholds. Approximately 70-80% of CpGs are included with a threshold of 5, while 30-50% are included in the analysis with a threshold of 10. (C-G) We reproduced our results using highly covered CpG sites (at least 10 reads), demonstrating that our results are not biased by CpG site read coverage. (C) Global (Weighted) DNA methylation levels of CG, CHG, and CHH in each sample show high levels of CpG methylation and low levels of CH methylation (comparable to Fig. 1A). (D) (Weighted) DNA methylation of promoters, gene bodies, and intergenic regions in 20 groups of genes with different expression levels, ranging from rank 0 to rank 20. A negative correlation is observed in promoters, while a bell-shaped correlation is seen in gene bodies (comparable to Fig. 1C). (E) Distributions of DNA methylation level differences at CpG sites between females and males in autosomes and the X chromosome show that the X chromosome is generally hypomethylated (comparable to Fig. 2B). (F) Differences between females and males in expression, promoter methylation, and gene body DNA methylation of genes across the X chromosome. Mann-Whitney U test p-value indicated. (comparable to Fig. 1D). (G) Fractional methylation levels of CpG sites around *Xist* are indicated, showing a female hypomethylated island near the 5’ end of the gene (comparable to Fig. 4B).
